# Depression Increases Stroke Hospitalization Cost: An Analysis of 17,010 Stroke Patients in 2008 by Race and Gender

**DOI:** 10.1155/2013/846732

**Published:** 2013-03-10

**Authors:** Baqar Husaini, Robert Levine, Linda Sharp, Van Cain, Meggan Novotny, Pamela Hull, Gail Orum, Zahid Samad, Uchechukwu Sampson, Majaz Moonis

**Affiliations:** ^1^Tennessee State University, Nashville, TN 37209, USA; ^2^Meharry Medical College, Nashville, TN 37208, USA; ^3^Harbor-UCLA Medical Center, Los Angeles, CA 90095, USA; ^4^Vanderbilt University, Nashville, TN 37203, USA; ^5^Charles R. Drew University, Los Angeles, CA 90059, USA; ^6^University of Massachusetts, Boston, MA 01655, USA

## Abstract

*Objective*. This analysis focuses on the effect of depression on the cost of hospitalization of stroke patients. *Methods*. Data on 17,010 stroke patients (primary diagnosis) were extracted from 2008 Tennessee Hospital Discharge Data System. Three groups of patients were compared: (1) stroke only (S^O^, *n* = 7,850), (2) stroke + depression (S^+D^, *n* = 3,965), and (3) stroke + other mental health diagnoses (S^+M^, *n* = 5,195). *Results*. Of all adult patients, 4.3% were diagnosed with stroke. Stroke was more prevalent among blacks than whites (4.5% versus 4.2%, *P* < 0.001) and among males than females (5.1% versus 3.7%, *P* < 0.001). Nearly one-quarter of stroke patients (23.3%) were diagnosed with depression/anxiety. Hospital stroke cost was higher among depressed stroke patients (S^+D^) compared to stroke only (S^O^) patients ($77,864 versus $47,790, *P* < 0.001), and among S^+D^, cost was higher for black males compared to white depressed males ($97,196 versus $88,115, *P* < 0.001). Similar racial trends in cost emerged among S^+D^ females. *Conclusion*. Depression in stroke patients is associated with increased hospitalization costs. Higher stroke cost among blacks may reflect the impact of comorbidities and the delay in care of serious health conditions. Attention to early detection of depression in stroke patients might reduce inpatient healthcare costs.

## 1. Background

Between 20% and 60% of stroke patients are diagnosed with depression/anxiety [1], and these are often newly diagnosed in stroke patients both during hospitalization and up to 3 years after discharge [1–20]. Depression is associated with longer institutionalization and poorer rehabilitation outcomes [21, 22]. Further, depression is more often diagnosed for females and white stroke patients [23, 24], and it is correlated with higher rates of suicidal ideation and stroke mortality [25–27]. Depression increases the risk of stroke [28] as well as increased healthcare costs [29–34]. As these and other stroke related factors are evaluated, understanding their impact on healthcare cost is necessary for better management, improved therapeutic outcomes, and reduced healthcare cost.

## 2. Depression and Healthcare Cost

Several studies have reported the effect of depression/anxiety on healthcare costs. For example, while female Medicare patients had a higher prevalence of depression and higher use of outpatient services, inpatient hospital costs for male patients were 47% higher compared to females ($15,060 versus $10,240, *P* < 0.001) [30]. In another study, the medical cost of depressed patients was 54% higher compared to nondepressed patients [34]. While higher cost among stroke patients is associated with greater number of readmissions, longer hospitalizations, and greater number of outpatient visits compared to a control group without depression, evidence is sparse about whether these costs vary by race and gender. 

In this study of Tennessee stroke patients (*n* = 17, 010), we examine two issues: (1) prevalence of depression among stroke patients by race and gender and (2) the effect of depression on total hospitalization cost in 2008 by race and gender.

## 3. Methods

### 3.1. Data

We obtained inpatient discharge data from the 2008 Tennessee Hospital Discharge Data System (HDDS) compiled by The Tennessee Department of Health's (TDH) Division of Health Statistics. All hospitals licensed by the TDH are required by law to report patient-level discharge information. Data are reported on a uniform billing form developed by the National Uniform Billing Committee. Diagnoses in the administrative files are given by the attending physicians (according to the ICD-9 codes), and it is unclear what tests are used in arriving at those diagnoses. Further, these diagnoses appear only when the patient is treated for those conditions in the hospital. We extracted data on primary diagnosis of stroke (ICD-9 codes of 430–438) along with the secondary diagnoses of depression/anxiety (ICD-9 codes 296.2—major depressive disorder, single episode, 292.3—major depressive disorder, recurrent episode, 3000.4—neurotic depression, 309.0—brief depressive reaction, 309.1—prolonged depressive reaction, 311—depressive disorder, not elsewhere classified, and 300—anxiety states, hysteria, phobic disorders, and neurotic depression) for blacks and whites since they constitute 97% of Tennessee population. Since there is a high overlap in symptoms of depression and anxiety ranging from 48% to 74% [37, 38], we combined the diagnoses for depression and anxiety as a single variable for our analysis. Data extraction on stroke patients included sex, age, race, days of hospitalization, number of re-admissions, and costs associated with stroke treatment as well as the total hospital charges for the entire year of 2008 when the patient was readmitted for illnesses other than stroke. Extracted data also included co-morbidities such as atrial fibrillation, hypertension, diabetes, cholesterol, and cardiovascular events such as heart attacks. The stroke sample included whites (82%) and females (55%), and the average age in the sample was 70 years. Stroke rates were age adjusted per 2000 US population.

### 3.2. Statistical Analysis

Analysis of variance compared the average hospitalization costs [39] for three groups of stroke patients: (1) stroke only (S^O^, *n* = 7, 850), (2) stroke + depression/anxiety (S^+D^, *n* = 3, 965), and (3) stroke + other mental diagnoses (S^+M^, *n* = 5, 195). The Fisher exact test was used for comparison of healthcare cost and prevalence of comorbidities by race and sex. Percentages of stroke diagnoses were compared using Pearson's Chi-squared test with Yates' correction for continuity, and odd ratios (ORs) were obtained through logistic regression analyses, which controlled for age, sex, hypertension, diabetes, cholesterol, and atrial fibrillation. A probability value of *P* < 0.05 was the accepted threshold for statistical significance.

## 4. Results

### 4.1. Prevalence of Stroke, Depression, Comorbidities, and Healthcare Cost

Our analysis showed that 17,010 patients (4.3% of all 400, 235 adult patients) had a primary diagnosis of stroke with an age-adjusted prevalence rate of 370.6 per 100 K. Stroke was higher among blacks compared to whites (4.5% versus 4.2%, resp., *P* < 0.0001; prevalence rates of 517.1 versus 322.0, resp.; OR = 1.31, 95% CI = 1.26–1.36 after controlling for risk factors, Table 1). Stroke was more prevalent among males than females (5.1% versus 3.7%, *P* < 0.0001; rates of 374.1 versus 369.2 per 100 K; OR = 1.22, 95% CI = 1.18–1.25). Further, stroke was more common among black males compared to white males (5.8% versus 5.0%, *P* < 0.001; prevalence rates of 532.3 versus 351.0 per 100 K; OR = 1.31, 95% CI = 1.23–1.39) and among black females compared to white females (3.9% versus 3.7%, *P* < 0.02; prevalence rates of 505.7 versus 298.9 per 100 K; OR = 1.29, 95% CI = 1.22–1.37). Nearly one-quarter of stroke patients were depressed/anxious (23.3%). Depression among stroke patients was higher among whites than blacks (25.1% versus 15.2%, *P* < 0.001) and among females than males (27.5% versus 18.3%, *P* < 0.001). 

Table 1 shows that nearly 20% (3,402 of 17,010) of stroke patients had congestive heart failure (CHF) and 4% had experienced heart attacks (MI). Coronary heart disease (CHD) was also more prevalent among the stroke patients (39% overall, 41% among whites versus 30% among blacks). Other stroke co-morbidities varied by race and sex. Hypertension (92%) and diabetes (45%) were more prevalent among blacks and atrial fibrillation (32%) and high cholesterol (17%) were more prevalent among white patients. 

Table 1 further shows that treatment cost associated with stroke only (S^o^) was higher among blacks as compared to whites ($41,370 versus $30,215, *P* < 0.001, a difference of 36.9%). This cost difference also exists when the average annual costs for the entire year of 2008 is examined for patients without stroke. Here again, nonstroke black patients compared to nonstroke white patients had higher 2008 cost ($45,892 versus $40,376, *P* < 0.001), partly due to longer hospitalization for black patients (8.6 days for blacks versus 7.3 days for whites, *P* < 0.001). The cost differential remained intact when the comparisons are made simply for stroke cost ($74,338 for blacks versus $55,884 for whites, *P* < 0.001) or the cost for the entire year of 2008 with multiple re-admissions ($74,338 for blacks versus $55,884 for whites, *P* < 0.001). Similar trends for stroke cost emerged for black males ($74,006 versus $59,403, *P* < 0.001) and for black females ($74,589 versus $52,877, *P* < 0.001). Again, higher costs among blacks reflected higher comorbidities and longer hospitalizations for blacks than whites. Thus, black stroke patients (due to high comorbidities and longer hospitalization) cost 62% more than nonstroke black peers ($74,338 versus $45,892, a difference of $28,446) and more than 80% of the cost for white nonstroke patients ($74,338 versus $40,376). Comparable race and gender differences also existed in costs associated with ischemic or hemorrhagic stroke (see Table 1). In summary, black patients had higher costs associated with hospitalizations compared to white patients no matter how the costs were examined.

### 4.2. Effect of Depression on Hospital Cost for Stroke

We examined cost and associated co-morbidities including Charlson Index of comorbidity for three stroke groups including: (1) patients with stroke only (S^O^); (2) patients with stroke + depression (S^+D^); (3) stroke patients with other mental diagnoses (S^+M^). Within each stroke category, we compared cost and associated factors by race and gender.  Table 2 shows that the average healthcare cost was nearly 63% higher for stroke patients with S^+D^ compared to S^O^ ($77,864 versus $47,790, *P* < 0.001, a difference of 63%) or S^+D^ compared to S^+M^ ($77,864 versus $62,387, *P* < 0.001, a difference of 24.8%). Clearly, these data show that depression among stroke patients is associated with higher hospital costs compared with stroke patients who have other mental illnesses.

Table 2 provides costs and comorbidities data for three groups of stroke patients, further illustrating that both stroke prevalence and annual costs were higher among blacks, and the race-sex differences are made evident. Among depressed stroke patients (S^+D^), black males had higher annual hospital charges compared to white males ($97,196 versus $88,115, *P* < 0.001), in part due to longer hospital stays compared to white males (24.6 versus 20.2, *P* < 0.001). Similarly, black S^+D^ females had higher cost compared to white S^+D^ females ($95,269 versus $68,184, *P* < 0.001). For black males, the higher cost cannot be attributed to depression/anxiety as only 11% of black males had a diagnosis of depression; the higher cost here appears to reflect complexities (denoted by a higher Charlson comorbidity index) that develop from co-morbid conditions such as higher prevalence of hypertension and diabetes. Similar race and gender trends also existed for black males and females across S^+M^ and S^O^ groups of patients. 

## 5. Comments

Previous studies on healthcare cost have reported substantially higher cost (54% higher) for patients with cardiovascular disease (CVD) and stroke in association with depression and anxiety [31–34]. Our analyses show that depression and anxiety among Tennessee stroke patients is associated with a 63% increase in the annual hospital care cost. Further, our findings of higher cost for depressed stroke patients, especially among women, are consistent with those reported previously [34]. Since depression can be considered as an independent risk factor for CVD [40, 41] and since women outnumber men in the population (as well as in our S^+D^ group—54% versus 46%), costs attributable to depression may be reduced by early diagnosis and treatment of depression. The stroke patients in our sample had higher prevalence of both hypertension (more than 80%) and diabetes (more than 35%). Addressing depression and reducing risk factors through preventive programs [42] could substantially reduce the morbidity, mortality, and healthcare costs associated with stroke [42, 43].

The average healthcare cost among blacks compared to whites were higher regardless of whether the stroke was hemorrhagic or ischemic (hemorrhagic cost—$64,643 versus $48,246 *P* < 0.001; ischemic cost—$41,120 versus $28,833, *P* < 0.001). These higher costs remained intact when total stroke costs (combined ischemic+hemorrhagic+unspecified stroke + TIA) were compared between blacks and whites ($41,370 versus $30,215, *P* < 0.001), particularly black males compared to white males ($41,586 versus $31,359, *P* < 0.001). The same cost pattern emerges when the annual cost for the entire 2008 year was combined (blacks had higher annual cost compared to whites: $74,338 versus $55,884, *P* < 0.001, Table 1) and when racial comparisons for nonstroke patients were made. These differences suggest that blacks with chronic conditions may seek medical services later in the progression of their disease and that this late entry to care [44, 45] may require more services and longer hospitalization as is evident in our data (16 days for black patients compared to 12 days for white patients, Table 1). Further, the higher cost among black males may in part exist because previous studies suggest that they are more likely to drop out of behavioral and pharmacological therapies [46] which in turn leads to more complications and readmissions (re-admissions are higher among blacks—see Table 1).

The lower overall cost of care among women (particularly white women) compared to men may result from a number of factors including that women, in general, seek professional help earlier on in the development of their illness compared to men [47, 48] and this alone may reduce complications and hence reduce length of hospitalization and cost [47–49]. In order to impact CVD end points among women, depression/anxiety must be treated both as independent risk factors for preventing CVD and for reducing cost in females with known CVD [50].

Finally, Our findings of higher hospitalization cost of stroke is associated with depression and anxiety that consistently appear as a co-morbid condition requiring greater attention in managing healthcare cost. Findings of higher cost and greater utilization of services, though scantly reported (see Table 3 below), nonetheless are supportive of monitoring ways to contain higher treatment cost associated with stroke and other major events.

## 6. Limitations

The administrative hospital discharge files do not provide clinical data regarding severity/duration of diseases, test results, or cost of pharmacological treatment provided. Further, these administrative files do not provide itemized cost, and hence it is impossible to determine the cost of pharmacological treatment for depression/anxiety for any patient. The administrative data only include the total cost for the entire hospital stay, number of admissions, and sometimes within the total cost per admission, the cost associated with major procedures such as CABG. In addition to the primary diagnosis, these administrative files provide data on secondary diagnoses (i.e., co-morbidities) only when treatment is provided for those conditions. These administrative files lack clinical details of diagnoses or co-morbid conditions which may shed additional light on racial and gender differences in healthcare cost. Our data are from a single state and for only one year (2008), and as such they may not reflect outcomes from other geographic areas/regions. Finally, based on this cross-sectional data, we were unable to differentiate prestroke depression from poststroke depression. However, in either case, the association of stroke with depression in our study seems to contribute to increased hospital stay, greater co-morbidities, and significantly greater cost of healthcare. 

## 7. Conclusion

 Stroke patients with depression/anxiety have significantly higher healthcare costs compared to those with stroke only (i.e., without depression/anxiety) or those with other mental health diagnoses. Based on the patterns reported here, greater attention to prevent comorbidities and early detection of depression in stroke patients are all promising interventions aimed at reducing inpatient healthcare costs while improving overall care, with the greatest opportunities for improved health and cost savings in the black male population. Analytic epidemiologic studies are needed to examine whether the higher healthcare costs among blacks exist due to delays in seeking treatment and/or poor access to services, leading to more complex problems and longer hospitalizations. Additionally, research is needed to determine whether aggressive treatment of depressed patients that have suffered stroke might reduce the overall costs of stroke care.

## Figures and Tables

**Table 1:** Age-adjusted stroke rates and characteristics by race and gender in 2008 (*n* = 17,010).

Variables	WF *n* = 7495	WM *n* = 6405	BF *n* = 1773	BM *n* = 1337	Total *n* = 17,010	Any stroke blacks *n* = 3110	Blacks no stroke *n* = 65,595	Any stroke whites *n* = 13,900	Whites no stroke *n* = 317,630
*Stroke rate per 100* K	298.9	351.0	505.7	532.3	370.6	517.1	—	322.0	—
Age	73	69	64	61	70	63	49	77	57
MI%	3.8	4.2	3.6	5.3^+^	4.1	4.3	3.1	4.0	4.4*
CHF%	19.5	17.9	20.1	21.3*	19.1	20.6*	14.5*	18.7	13.6
CHD%	31.7*	31.4	23.6	24.3	38.9	29.8	17.5	41.0	25.8*
At fib%	32*	31	24	24	30	24	13.0	32*	18.6*
Hyp%	85	84	92	92*	86	92*	54	84.5	52
Diabetes%	31	36	47*	42	36	45*	27.4	34	22.7
Chol%	15.8	17.3*	11.3	13.4	15.7	12.2	5.1	16.5*	8.2*
Dep/anx%	29.7*	19.8	18.3	11.0	23.3	15.2	10.7	25.1*	21.4*
Number of readmissions	1.11	1.12	1.13	1.13	1.12	1.13	1.6	1.11	1.5
Hop stk day	5.0	5.1	7.1	7.3	5.4	7.2*	—	5.0	—
Tot hos day	11.3	11.7	15.7	15.4	12.2	15.6*	8.6*	11.5	7.3
Average ischemic $	27,071	30,904	43,074*	38,710	31,460	41,120*	—	28,833	—
Average hemo. $	45,852	50,017	69,796*	60,586	51,211	64,643*	—	48,246	—
Average Total stroke $^+^	29,238	31,359	41,207	41,586*	32,255	41,370*	—	30,215	—
All 2008 admis $	52,877	59,403	74,589	74,006	59,259	74,338	45,892*	55,884	40,376

^+^Average cost for all strokes combined includes cost associated with ischemic + hemorrhagic + unspecified strokes + TIA; # is average number of admissions/hospital days; CHF: congestive heart failure; CHD: coronary heart disease; all costs are reported in averages. Average of all total 2008 cost includes cost combined for all admissions in 2008.

*Fisher's exact test differences are significant between nonstroke black and white patients at *P* < 0.001.

**Table 2:** Three stroke group cost by race and gender, 2008.

	Stroke only S°, *n* = 7,850; age = 71; entire 2008 cost = $47,790				Stroke + Dep S^+D^, *n* = 3,965; age = 68; entire 2008 cost = $77,864*				Stroke + other ment S^+M^, *n* = 5,195; age = 69; entire 2008 cost = $62,387			
	WF	WM	BF	BM	WF	WM	BF	BM	WF	WM	BF	BM
Mean age	75	71	64	61	71	67	62	61	73	67	65	61
HTN%	84	83	91	91	87	89	96	93	83	83	91	92
Diabetes%	31	36	48	47	34	42	55	48	30	32	42	36
CHF%	17	15	17	21	23	25	26	29	20	19	22	20
MI%	2.9	3.3	3.4	3.5	4.4	6.5	3.4	7.5	5.0	4.2	4.1	6.4
Atrial fibrillation	31*	30	22	23	32	36	24	31	32*	30	26	24
Hospital days	8.1	7.8	12.7	12.8	15.7	20.2	21.9	24.6	11.6	12.2	16.9	15.6
Number of Admissions	1.7	1.7	1.9	1.8	3.0	3.6	2.9	3.2	2.2	2.1	2.3	2.3
Comorb Index^++^	1.4	1.5	1.7	1.9*	1.6	1.9	2.1	2.3*	1.7	1.8	1.9*	1.7
Total stroke cost combined $	27,601	28,926	39,500	40,866*	31,369	32,488	39,482*	37,773	29,630	34,209	44,914*	43,140
Annual cost for all 2008 admissions $	42,329	46,210	63,072	64,622*	68,184	88,115	95,269	97,196*	53,575	61,155	80,849*	77,076

*Differences significant at *P* < 0.001; ^++^Charlson Comorbidity Index—higher score denote greater number of comorbid conditions.

**Table 3:** Increased medical care costs of stroke associated with depression. Recent peer-reviewed publications.

First author and year	Country	Type of study	Participants	Results	Conclusions
Bhattarai et al., [35] 2012	UK	Population-based cohort	299,912 participants, ages 30 to 100 years	14% of male and 26% of female stroke patients with single morbidity had comorbid depression; patients with concurrent diabetes, CHD, and stroke had a very high prevalence of depression (men 23% and women 49%)	Compared to those with no morbidity, depression was associated with higher rates of healthcare utilization and increased costs at any level of morbidity.

Sicras et al., [36] 2008	Spain	Cross-sectional, retrospective	2,266 stroke patients	Females (OR 2.1), obesity (OR 1.1), and neuropathy (OR 2.2) were significantly associated with depressive disorder in stroke patients	Adjusted total costs of depressive disorder were higher in most components, euro 2, −37.55 versus euro 1,498.24 (*P* < 0.001). Medication drugs accounted for 73.4% of the total costs.

Jia et al., [29] 2006	USA	National cohort	5,825 Department of Veterans Affairs patients with stroke	41% of the sample had poststroke depression	After adjusting for patient demographic and clinical factors, patients with stroke and poststroke depression had significantly *P* < 0.0001, more hospitalization, outpatient visits, and longer length of stays, 12 months after stroke compared with patients with stroke but no poststroke depression
